# A dual-task gait test detects mild cognitive impairment with a specificity of 91.2%

**DOI:** 10.3389/fnins.2022.1100642

**Published:** 2023-02-07

**Authors:** Yuxin Wang, Qing Yang, Chong Tian, Jing Zeng, Mengshu Yang, Jie Li, Jing Mao

**Affiliations:** ^1^School of Nursing, Tongji Medical College, Huazhong University of Science and Technology, Wuhan, Hubei, China; ^2^Department of Nursing, Tongji Hospital, Tongji Medical College, Huazhong University of Science and Technology, Wuhan, Hubei, China; ^3^Centers for Disease Control and Prevention of Wuhan Economic and Technological Development Zone, Wuhan, Hubei, China

**Keywords:** mild cognitive impairment, gait, dual-task, older adults, AniP-DT

## Abstract

**Background:**

Mild cognitive impairment (MCI) is a valuable intervention window in the progress of senile dementia, but the question of how to easily and conveniently detect MCI in the community remains unanswered. Gait performance reflects cognitive function, but how to reliably detect MCI through gait testing is still being explored.

**Objective:**

To develop a dual-task gait testing method that could reliably detect MCI in the community.

**Methods:**

A cross-sectional diagnostic study was conducted in 111 older adults (mean age = 72.14 ± 6.90 years) from five communities in Wuhan, China. A novel dual-task gait testing method, walking while identifying animals in pictures (AniP-DT gait test), was developed. The participants were classified into MCI or cognitively intact based on their performance on the Montreal Cognitive Assessment Scale (MoCA). Gait performance was assessed using both single-task and the AniP-DT gait test. Multiple linear regression and binary logistic regression were used to model the association between gait speed and cognitive status, and receiver operating characteristic (ROC) curve analysis was used to assess the discrimination ability.

**Results:**

Compared to the cognitively intact group, the gait speed of the MCI group was lower in both single-task and the AniP-DT gait tests. The gait speed of the AniP-DT gait test was significantly associated with MoCA scores after adjusting the covariates and exhibited good discrimination ability in MCI detection (AUC = 0.814), with a specificity of 91.2%. ROC analysis of the logistic models revealed better discrimination ability of dual-task gait velocity when adjusted with age and years of education (AUC = 0.862).

**Conclusion:**

The evidence in this study suggested that the AniP-DT gait test could be an easy and reliable screening tool for MCI in community older adults.

## Introduction

Dementia is a devastating condition carrying huge social and healthcare burdens (Alzheimer’s and Dementia, 2021), and will increase with the population aging. Without effective treatment for dementia, mild cognitive impairment (MCI), a transitional stage between normal aging and dementia that can be treated and even reverted (Petersen et al., 2018), becomes a valuable window to intervene in the progress of dementia. However, symptoms of MCI are often subtle and few patients seek medical assistance before their condition worsens, Thus, identifying MCI patients in the community population would be a precondition for the MCI intervention. The current screening methods for MCI are mostly for clinical settings (Connolly et al., 2011), and effective and convenient community screening tool for MCI is still in short supply.

Motor markers of cognitive changes have gained growing attention in recent years (Montero-Odasso et al., 2009; Beauchet et al., 2013). People with cognitive impairment often demonstrate gait disturbances, and the underlying mechanism points to brain regions and networks shared between gait motor control and cognitive processes (Montero-Odasso et al., 2014). A slowing in gait velocity was found associated with cognitive impairment and progression to dementia (Montero-Odasso et al., 2005; Waite et al., 2005). Moreover, studies have suggested that gait disturbances may precede the evident cognitive symptoms in dementia (Buracchio et al., 2010; Kikkert et al., 2016). Therefore, gait tests, characterized by low cost and convenience, could be a potential screening tool for cognitive impairment. A seminal study in 1997 stated that older adults who stopped walking while talking had a higher risk of falls (Lundin-Olsson et al., 1997). Since then, the dual-task gait test, a test in which participants perform walking and a secondary cognitive task concurrently (Al-Yahya et al., 2011), has become a research topic to explore the relationship between gait and cognition. Our recent meta-analysis demonstrated that the dual-task gait test exhibited higher sensitivity in MCI detection compared with a simple walking test (Yang et al., 2020). Because of the limited attentional capacity in people with compromised cognitive abilities, interference in the performance of one or both tasks would occur when an extra task was added (Jayakody et al., 2020).

There are various forms of dual-tasking and many cognitive tasks have been used in the dual-task paradigms. These tasks differentiate from each other in their nature, content and task difficulty. More importantly, different cognitive tasks have shown varying effects in people with MCI (Hunter et al., 2018). Currently, there is no consensus about which cognitive task should be paired with walking or which has a better discriminating ability for MCI (Montero-Odasso et al., 2019). According to Al-Yahya et al. (2011), cognitive tasks can be divided into five categories: reaction time, discrimination and decision-making, mental tracking, working memory, and verbal fluency, which relate to different cognitive domains. Several tests are commonly used in dual-tasks for older adults with MCI, including serial subtractions by 1 or 7 (mental tracking) (Montero-Odasso et al., 2017; Cullen et al., 2018; Hunter et al., 2018), naming animals (verbal fluency) (Montero-Odasso et al., 2017; Cullen et al., 2018; Hunter et al., 2018), and delayed recall (working memory) (Tseng et al., 2014; Nascimbeni et al., 2015). Discrimination and decision-making tests, however, are rarely used. Studies demonstrated that increasing the complexity of the cognitive task could bring greater sensitivity for MCI detection (Bahureksa et al., 2017; Yang et al., 2020). However, raising the difficulty too much could hurt specificity. The task needs to be challenging enough so that participants are working at or near the limit of their ability to uncover deficits (Hunter et al., 2018). On the other hand, MCI patients could exhibit impairment in different cognitive domains, including executive function, attention, language, memory, and visuospatial skills (Petersen et al., 2014). People always show different proficiency in different domains, so only testing one domain could conceal the deficit of other domains. Thus, developing a comprehensive task with appropriate complexity would improve the discrimination ability of the task greatly.

Picture naming tasks, which belong to the discrimination and decision-making tasks, are wildly used to characterize cognitive impairment. This task involved the function of visual perception, semantic processing, word retrieval, and oral naming, containing multiple cognitive processes (Lin et al., 2014). The multiple processes could test multiple cognitive domains (semantic memory, executive function, language, attention). In this study, we chose animal pictures because people are reasonably well knowledgeable about animal names; hence the influence of gender, age, and education could be minimal. In this way, we have developed an AniP-DT gait test, pairing the animal picture naming task with normal walking to form a dual-task gait test. The animal picture naming task required the participants to discriminate and name the animals in pictures, impairment in any domain (visual perception, semantic processing, word retrieval, and oral naming) could result in interference in motor performance. Therefore, using the AniP-DT gait test to assess motor-cognitive interaction would detect mild impairment in different cognitive processes, and improve the MCI detection capabilities.

## Materials and methods

### Study design, setting, participants

This study followed the strengthening of the reporting of observational studies in epidemiology (STROBE) guidelines. A cross-sectional diagnostic study was conducted in five communities in Wuhan, China from March to July 2019. Inclusion criteria included: (1) older adults aged 60 years old and above; (2) able to follow test instructions; (3) normal vision and hearing; (4) able to walk 10 m independently (assistive walking devices including canes and walkers were allowed). Subjects were excluded if they had: (1) musculoskeletal disorders of lower limbs that affect gait performance (e.g., arthritis); (2) central or peripheral neurological diseases (e.g., Parkinsonism, stroke); (3) recent acute illness or surgery (in the past 3 months) (Nascimbeni et al., 2015). The study protocol was approved by the responsible institutional review board. Informed consent was obtained from each participant before data collection. All data has been de-identified and used only for academic purposes.

### Medical and cognitive assessments

Sociodemographic information and comorbidities were collected. Sex, age, body mass index (BMI), years of education, smoking, alcohol drinking, and use of walking aids were collected as sociodemographic characteristics. BMI is a person’s weight (kg) divided by the square of height (m). Current smokers were participants who smoked at least one cigarette per day for over 6 months. Current alcohol drinkers were defined as drinking alcohol every day for more than 6 months. Cognition was assessed using the Montreal Cognitive Assessment Scale (MoCA) (Nasreddine et al., 2005). The score ranges from 0 to 30, and a higher score indicates better cognitive performance. MoCA has high sensitivity and reliability to screen MCI patients. A Chinese version of MoCA was used and adjusted with educational level (one correctional point was given to participants with less than 12 years of education except for those who already scored 30). Participants with MoCA scores less than 24 were categorized as MCI in this study. Others were classified as cognitively intact.

### Psychological assessments

Previous studies have reported neuropsychiatric symptoms in cognitive decline individuals (Somme et al., 2013). Older adults’ depression, anxiety, and apathy status were recorded in this study. The Chinese version of the geriatric depression scale with 15 items (GDS-15) was used to assess the depressive symptoms and a score of 8 or above indicated depression (Zhao et al., 2019). Anxiety was evaluated using the Chinese version of the geriatric anxiety inventory (GAI) (Yan et al., 2014). A score greater than 10 indicated anxiety. Apathy was assessed with the apathy evaluation scale-self (AES-s) (Marin et al., 1991). In all three scales, higher scores indicated severer psychological problems.

### Gait assessments

**FIGURE 1:**
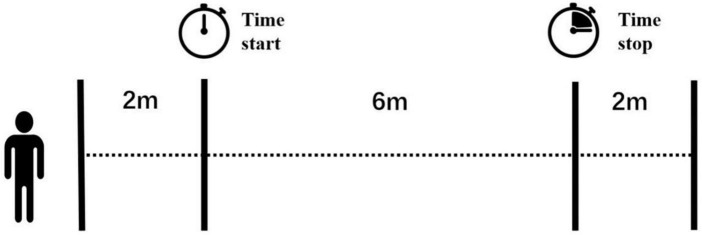
View of the 10-m gait test. Four lines of tape were marked on the ground and the two lines in the middle indicated the start and stop of a handheld stopwatch. The examiner started or stopped the timer once the participant’s first foot passed these two lines.

Gait velocity of self-selected ground walking was recorded using a 10-meter-walk test. In each community, participants performed the tests in a well-lit, dry and spacious area, eliminating any dangerous objects from the ground. We adopted a stopwatch and tape measure method (Youdas et al., 2010). Four lines of tape were used on the ground to mark the start line, 2-m line, 8-m line, and finish line (Figure 1). The duration of each test was measured using a stopwatch, with a measurement error of 0.01 s.

While participants needed to walk through the 10-m distance, the measurement distance was in the middle, at 6-m. The examiner started the timer once the participant’s first foot crossed the 2-m line and stopped the timer when the participant’s first foot crossed the 8-m line. In this way, we could measure the steady gait performance without the acceleration and deceleration phases. Older adults were given rest between each trial if needed to reduce the effect of fatigue. The examiner walked slightly behind (out of their field of vision) to protect them from falling. No adverse events happened during the tests.

Each participant performed four walks, in the order of two single-task tests and two dual-task tests. In the single-task tests, older adults were instructed to walk at their preferred speed. In the dual-task tests, the cognitive task was animal picture naming. Older adults were required to name the animals in the pictures printed on paper while walking at their usual speed. The paper was A4 size and on each side, nine photographs of animals were printed, including both common (e.g., cat, rabbit) and low-familiarity animals (e.g., hedgehog, camel) for Chinese older adults (Supplementary Figure 1). The paper was given to participants right before the AniP-DT gait tests. All participants were given the same instructions to assure consistency and were not asked to prioritize any task over another. They had no practice trials. Gait velocities (cm/s) of single-tasks and dual-tasks were averaged from the two trials, respectively.

### Data analysis

Sociodemographic, clinical, and gait characteristics were presented using either means and standard deviations (SD) or frequencies and percentages. Participants were divided into two groups (cognitively intact and MCI) based on the MoCA scores. Comparisons between the two groups were made using *t*-tests and Chi-square tests as appropriate. Spearman’s rank correlation was used to explore the universal correlation between gait parameters and MoCA scores. Then we used multiple linear regression models that included the MoCA score as a dependent variable and the gait parameters, adjusted for the potential confounders, as independent variables. Binary logistic regression was performed using MCI as the dependent variable and gait performances, adjusted for age and years of education as the independent variables. Next, we applied receiver operating characteristic (ROC) analysis to explore the discrimination ability of the gait performances for screening MCI. The cut-off value of gait speeds was determined using the Youden index. All statistical analyses were implemented using SPSS v.22 (IBM, NY, USA), with statistical significance set at *P* < 0.05 (2-sided).

## Results

### Characteristics and gait performance of participants

In total, 114 older adults participated in the study, of whom 3 dropped out because they could only perform the single-task tests due to fatigue. Finally, 111 participants were included in the analysis. The mean age was 72.14 ± 6.90 years and 51.4% were male. Participants’ characteristics, stratified by cognitive status, were presented in Table 1. There were 43 (38.7%) older adults classified as MCI. Participants with MCI were older and had fewer years of education. Also, older adults with MCI had a higher level of apathy.

**TABLE 1:** Characteristics of participants stratified by cognitive status.

	Full sample (*n* = 111)	Cognitively intact (*n* = 68)	MCI (based on MoCA) (*n* = 43)	*P*-value
Age, mean (SD), years	72.14 (6.90)	70.21 (6.08)	75.21 (7.08)	<0.001*
Male, n (%)	57 (51.4)	40 (58.8)	17 (39.5)	0.048*
BMI, mean (SD), kg/m^2^	23.17 (3.49)	23.40 (2.94)	22.81 (4.23)	0.42
Years of education, mean (SD)	10.08 (4.51)	11.46 (3.41)	7.91 (5.16)	<0.001*
Use of walking aid, n (%)	9 (8.1)	5 (7.4)	4 (9.3)	0.99
Current smoker, n (%)	21 (18.9)	12 (17.6)	9 (20.9)	0.67
Current alcohol drinker, n (%)	24 (21.6)	15 (22.1)	9 (20.9)	0.89
**Comorbidities, n (%)**				
Hypertension	48 (43.2)	31 (45.6)	17 (39.5)	0.53
Diabetes	16 (14.4)	13 (19.1)	3 (7.0)	0.08
Hyperlipidemia	7 (6.3)	5 (7.4)	2 (4.7)	0.86
GDS-15 (depression), mean (SD)	3.38 (3.09)	3.07 (2.96)	3.86 (3.26)	0.20
GAI (anxiety), mean (SD)	2.65 (3.51)	2.29 (2.98)	3.21 (4.18)	0.18
AES-s (apathy), mean (SD)	30.23 (9.06)	28.47 (8.33)	33.00 (9.55)	0.010*

MCI, mild cognitive impairment; BMI, body mass index; GDS, geriatric depression scale; GAI, geriatric anxiety inventory; AES-s, apathy evaluation scale-self; SD, standard deviation; MoCA, Montreal cognitive assessment.

*P*-values are shown for differences between cognitively intact and MCI using *t*-tests or chi-square tests.

*Statistically significant value. MCI was based on the MoCA score.

Table 2 showed the cognitive and gait performances of participants. As expected, the MCI group had a significantly lower MoCA score than the control group. Gait velocities of AniP-DT tests in both groups were much lower than those in the single-task tests. Participants with MCI had significantly lower gait velocity in both of the tasks, with more slowing in the dual task.

**TABLE 2:** Cognitive and gait performance of participants.

	Full sample (*n* = 111)	Cognitively intact (*n* = 68)	MCI (based on MoCA) (*n* = 43)	*P*-value
MoCA, median (range)	25 (12–30)	26 (24–30)	19 (12–23)	<0.001*
Single-task gait velocity, mean (SD), cm/s	117.22 (27.50)	125.47 (26.20)	104.16 (24.52)	<0.001*
AniP-DT gait velocity, mean (SD), cm/s	82.54 (27.65)	92.80 (25.21)	66.31 (23.41)	<0.001*

MCI, mild cognitive impairment; SD, standard deviation; MoCA, Montreal cognitive assessment; AniP-DT, animal picture naming dual-task.

*P*-values are shown for differences between cognitively intact and MCI using *t*-tests or chi-square tests.

*Statistically significant value.

MCI was based on the MoCA score.

### Associations between participants’ cognition and gait parameters

According to the spearman correlation tests, gait velocities of both single and dual-task were positively associated with the MoCA scores (single-task gait velocity, ρ = 0.406, *p* < 0.001; AniP-DT gait velocity, ρ = 0.566, *p* < 0.001). Table 3 presented the multiple linear regression analysis results. Each analysis was adjusted with age, sex, educational level, smoking and drinking status, use of walking aid, and psychological parameters. The results also showed that both of the gait velocities were significantly correlated with MoCA, and AniP-DT gait velocity could better account for the cognition of older adults. Table 4 reported the two logistic regression models regarding gait velocities and MCI. Controlling for age and years of education, participants who had lower dual-task (OR = 0.964, 95% CI = 0.941–0.988, *p* = 0.004) and single-task gait speeds (OR = 0.976, 95% CI = 0.957–0.997, *p* = 0.023) were more likely to have MCI.

**TABLE 3:** Results of multiple linear regression regarding the relationship between gait velocity and MoCA score.

Dependent variable	Independent variable	Coefficient	Standard error	*P*-value	*R* ^2^	*R*^2^ adjusted
MoCA	Single-task gait velocity	0.176	0.013	0.033*	0.471	0.418
	AniP-DT gait velocity	0.301	0.013	0.001*	0.509	0.460

MoCA, Montreal cognitive assessment; AniP-DT, animal picture naming dual-task. The linear regression model was adjusted with age, sex, educational level, smoking and drinking status, use of walking aid, and psychological parameters.

*Statistically significant value.

**TABLE 4:** Logistic regression models regarding gait velocities and MCI.

	Variable	OR	95% CI of OR	*P*-value
Model 1	AniP-DT gait velocity	0.964	0.941–0.988	0.004*
	Age	1.119	1.027–1.0240	0.018*
	Years of education	0.757	0.648–0.885	0.001*
Model 2	Single-task gait velocity	0.976	0.957–0.997	0.023*
	Age	1.153	1.059–1.255	0.001*
	Years of education	0.786	0.689–0.897	<0.001*

OR, odds ratio; CI, confidence interval; MCI, mild cognitive impairment; MoCA, Montreal cognitive assessment; AniP-DT, animal picture naming dual-task.

*Statistically significant value.

MCI was based on the MoCA score.

### Discrimination performance of gait speeds for screening MCI

**FIGURE 2:**
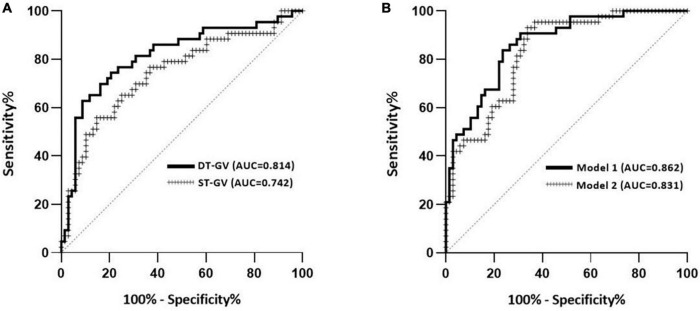
Discrimination performance of the gait parameters for screening MCI. **(A)** Receiver-operating characteristic (ROC) curves and corresponding area under the curve (AUC) for the dual-task (DT) gait velocity and single-task (ST) gait velocity to separate older adults with MCI in our study population. **(B)** ROC curves and corresponding AUC of two logistic models in Table 3. Model 1 contained DT gait velocity, age, and years of education. Model 2 contained ST gait velocity, age, and years of education. MCI was based on the MoCA score.

Figure 2 showed the receiver-operating characteristic (ROC) curves and area under the curve (AUC) of single and dual-task gait velocity for screening MCI. The AUC of AniP-DT gait velocity was 0.814 (95% CI = 0.727–0.900; *p* < 0.001), higher than that of single-task gait speed. The cut-off point of AniP-DT gait velocity was 65.78 cm/s (sensitivity = 0.628, 1–specificity = 0.088), and for the corresponding ROC curves of the two logistic models, the AUC of model 1 (0.862; 95% CI = 0.794–0.929; *p* < 0.001) was higher than model 2 (0.831; 95% CI = 0.757–0.905; *p* < 0.001), and improved when compared with only AniP-DT gait velocity (0.814).

## Discussion

This study developed a novel dual-task gait test (AniP-DT) that contained an animal picture naming task to test the MCI detecting ability in Chinese older adults. The MCI group had deteriorated gait performance compared to the cognitively intact group in both single and dual-task. Both single and dual-task gait velocities were significantly associated with MoCA scores even after adjustment of covariates. AniP-DT gait speed exhibited higher specificity in MCI detection in older adults. We found older adults whose AniP-DT gait velocity was less than 65.78 cm/s could be considered to have possible MCI in community settings. Adjustion of age and education could further improve the discrimination ability of AniP-DT gait speed. Our results suggested that gait velocity in AniP-DT could act as a behavioral marker to detect MCI.

Researchers have attempted to develop other motor-cognitive dual-task tests to help detect cognitive impairment (Klotzbier and Schott, 2017; Nielsen et al., 2018; Osuka et al., 2020). For instance, Latorre Román et al. (2020) designed a complex gait test (CGT) which was conducted on a 4 m × 6 m ground area and involved several obstacles when participants were walking. Among the discrimination and decision-making tasks, Trail-Walking Task (TWT) has also been used in MCI detection. Klotzbier and Schott (2017) used three TWT conditions with increased difficulty in a 4 m × 4 m area to differentiate MCI from cognitively healthy controls. Considering that a screening tool should be reliable, easy to use, and applicable to as many people as possible, we chose straight-path walking as the motor task, which has the lowest environmental and professional requirements, and chose animal picture naming as the cognitive task to ensure the scope of application for people are reasonably well knowledgeable about animals. The animals chosen in our picture naming test are familiar to most Chinese people. The whole test paradigm could be performed in a variety of settings including hospital hallways, local community centers, and parks, by personnel with simple training. On average, the test time for one person is less than 3 min.

In line with the previous findings (Montero-Odasso et al., 2014; Tseng et al., 2014), dual-task gait speed slowing was more prominent in our study and AniP-DT gait speed showed great discrimination ability of MCI from cognitively intact controls. The results suggested that the AniP-DT gait test performed well in MCI detection (AUC = 0.814), with a sensitivity of 62.8% and a specificity of 91.2%. In the complex gait test (CGT) of Latorre Román et al. (2020), obstacle negotiation could test participants’ executive function and exhibit a good diagnostic ability of MCI (AUC = 0.768). In the physical function test of Abe et al. (2022), models incorporating gait speed also showed good distinguish efficacy for MCI (AUC = 0.79), with a sensitivity of 73% and a specificity of 70%. The results from Klotzbier et al. showed that one of the TWT tests, the TWT-3, which has the highest cognitive load, showed a better differentiation (AUC = 0.860), the sensitivity of this task reached 100%, and the specificity was 66.67%. With a relatively close differentiation (AUC 0.814 vs. AUC 0.860), the sensitivity (62.8 vs. 100%) and specificity (91.2 vs. 66.67%) in our test and the results from Klotzbier et al. were nearly opposite. This could be due to the increased complexity of the task since TWT-3 involved participants’ cognitive flexibility, inhibition, and working memory (Klotzbier and Schott, 2017). Due to the stigma attached to dementia and cognitive impairment, screening and diagnostic decisions should be made with caution, thus particularly notable within this study is the high specificity of this test, which would be valuable in the detection of cognitive impairment in a large population. Further, we have determined the cut-off value of the gait speed. Older adults who walked slower than the cut-off value should have further evaluation of their cognition.

In widely used cognitive scales like MMSE or MoCA, age and years of education are often considered as the two factors greatly affect the performance of the participants. Xie et al. (2019) recruited MCI and cognitively normal participants in the community with matched demographic information (age, gender and education level). When adding these two parameters in our models, the ROC in our models also showed improved results, confirming the necessity of considering education and age in cognitive assessments. We think that the reason why the ajustment of age improved the differentiation ability of our test is that it is commensurate that gait velocity decreases with age, while the adjustment of education years increased the AUC of our models because we used the results from a Chinese version of MoCA, which is adjusted with educational level, as the reference in this study.

### Study limitations

There are several limitations to this study. We did not make a confirmed diagnosis of cognitive impairment with the Clinical Diagnosis (DSM-5) as a criterion but used MoCA results for the categorization of the participants, which might have brought in unknown confounding factors related to MoCA. Meanwhile, several approaches could further improve the AniP-DT paradigm. First, the current test has high specificity and moderate sensitivity when detecting MCI, implying that we could modify the difficulty of the cognitive task, hence, the animal picture naming task. Possible solutions could be changing the picture sequence by putting similar animals together to increase confusion or adding more low-familiarity animals. Secondly, we only included gait velocity as an outcome. Other parameters, including gait parameters (such as gait variability or cadence) and cognitive performance (number of correct animals, error type), could be tested for their discrimination abilities. Lastly, screening methods for MCI based on gait performance are mainly focused on Western countries with fewer studies in Eastern countries. Also, future studies aiming to upgrade and validate this task in larger samples and different regions are needed due to the existence of geographical cultural differences between Eastern and Western populations.

## Conclusion and implications

In summary, the AniP-DT gait test, which combined animal picture naming and straight-path walking, exhibited good discrimination ability in MCI detection in Chinese older adults in the community with a cutoff point of 65.78 cm/s. Although further exploration in a larger population is still needed, the testing paradigm showed great potential as a community screening tool for MCI in older adults, which could help identify MCI patients, thereby preventing or delaying their progression into dementia.

## Data availability statement

The raw data supporting the conclusions of this article will be made available by the authors, without undue reservation.

## Ethics statement

The studies involving human participants were reviewed and approved by the Medical Ethics Committee of Tongji Medical College, Huazhong University of Science and Technology, Wuhan, Hubei, China (s906). The patients/participants provided their written informed consent to participate in this study.

## Author contributions

QY, YW, CT, JZ, and MY: acquisition of data. YW, QY, CT, MY, and JL: analysis and interpretation of data. YW, QY, and CT: drafting of the manuscript. All authors contributed to study conception and design, critical revision of the manuscript for important intellectual content, and approved the submitted version.
