# Supplementary material for: A dual-task gait test detects mild cognitive impairment with a specificity of 91.2%
Source: Front Neurosci. 2023 Feb 7;16:1100642. doi: 10.3389/fnins.2022.1100642 (PMC9942944; doi:10.3389/fnins.2022.1100642)
Supplement: Supplementary file 1 [file Table_1.docx]

**Results of multiple linear regression regarding the relationship between single-task gait velocity and MoCA score (Model 1)**

| Model 1 Summary | R | R^2^ | R^2^-adjusted | Standard error | P-value | n |
| --- | --- | --- | --- | --- | --- | --- |
|  | 0.687 | 0.471 | 0.418 | 3.339 | ＜0.001 | 111 |
| Contributing factors | B | Standard error | β | t | P-value | VIF |
| **Single-task gait velocity** | 0.028 | 0.013 | 0.176 | 2.165 | 0.033 | 1.251 |
| Age | -0.257 | 0.052 | -0.405 | -4.969 | 0.000 | 1.259 |
| Sex | 1.277 | 0.751 | 0.146 | 1.699 | 0.092 | 1.403 |
| Years of education | 0.334 | 0.079 | 0.343 | 4.233 | 0.000 | 1.245 |
| Currently smoking | -2.623 | 1.063 | -0.236 | -2.467 | 0.015 | 1.727 |
| Currently drinking | 0.869 | 0.982 | 0.082 | 0.885 | 0.378 | 1.625 |
| Use of walking aid | 1.378 | 1.216 | 0.086 | 1.133 | 0.260 | 1.097 |
| GDS-15(depression) | -2.657 | 1.187 | -0.202 | -2.239 | 0.027 | 1.546 |
| GAI (anxiety) | -2.707 | 1.741 | -0.129 | -1.555 | 0.123 | 1.298 |
| AES-s(apathy) | -0.072 | 0.038 | -0.148 | -1.872 | 0.064 | 1.185 |

**Results of multiple linear regression regarding the relationship between AniP-DT gait velocity and MoCA score (Model 2)**

| Model 2 Summary | R | R^2^ | R^2^-adjusted | Standard error | P-value | n |
| --- | --- | --- | --- | --- | --- | --- |
|  | 0.713 | 0.509 | 0.460 | 3.218 | ＜0.001* | 111 |
| Contributing factors | B | Standard error | β | t | P-value | VIF |
| **AniP-DT gait velocity** | 0.048 | 0.013 | 0.301 | 3.564 | 0.001 | 1.453 |
| Age | -0.218 | 0.052 | -0.343 | -4.201 | 0.000 | 1.358 |
| Sex | 0.961 | 0.732 | 0.110 | 1.314 | 0.192 | 1.433 |
| Years of education | 0.337 | 0.075 | 0.347 | 4.498 | 0.000 | 1.214 |
| Currently smoking | -2.877 | 1.022 | -0.259 | -2.816 | 0.006 | 1.717 |
| Currently drinking | 1.354 | 0.961 | 0.128 | 1.409 | 0.162 | 1.679 |
| Use of walking aid | 1.797 | 1.178 | 0.113 | 1.525 | 0.130 | 1.108 |
| GDS-15(depression) | -2.780 | 1.145 | -0.212 | -2.428 | 0.017 | 1.548 |
| GAI (anxiety) | -2.522 | 1.676 | -0.120 | -1.504 | 0.136 | 1.295 |
| AES-s(apathy) | -0.054 | 0.037 | -0.112 | -1.457 | 0.148 | 1.209 |
