# Supplementary material for: A dual-task gait test detects mild cognitive impairment with a specificity of 91.2%
Source: Front Neurosci. 2023 Feb 7;16:1100642. doi: 10.3389/fnins.2022.1100642 (PMC9942944; doi:10.3389/fnins.2022.1100642)
Supplement: Supplementary file 2 [file Data_Sheet_1.docx]

Supplementary Material

## Supplementary Figure


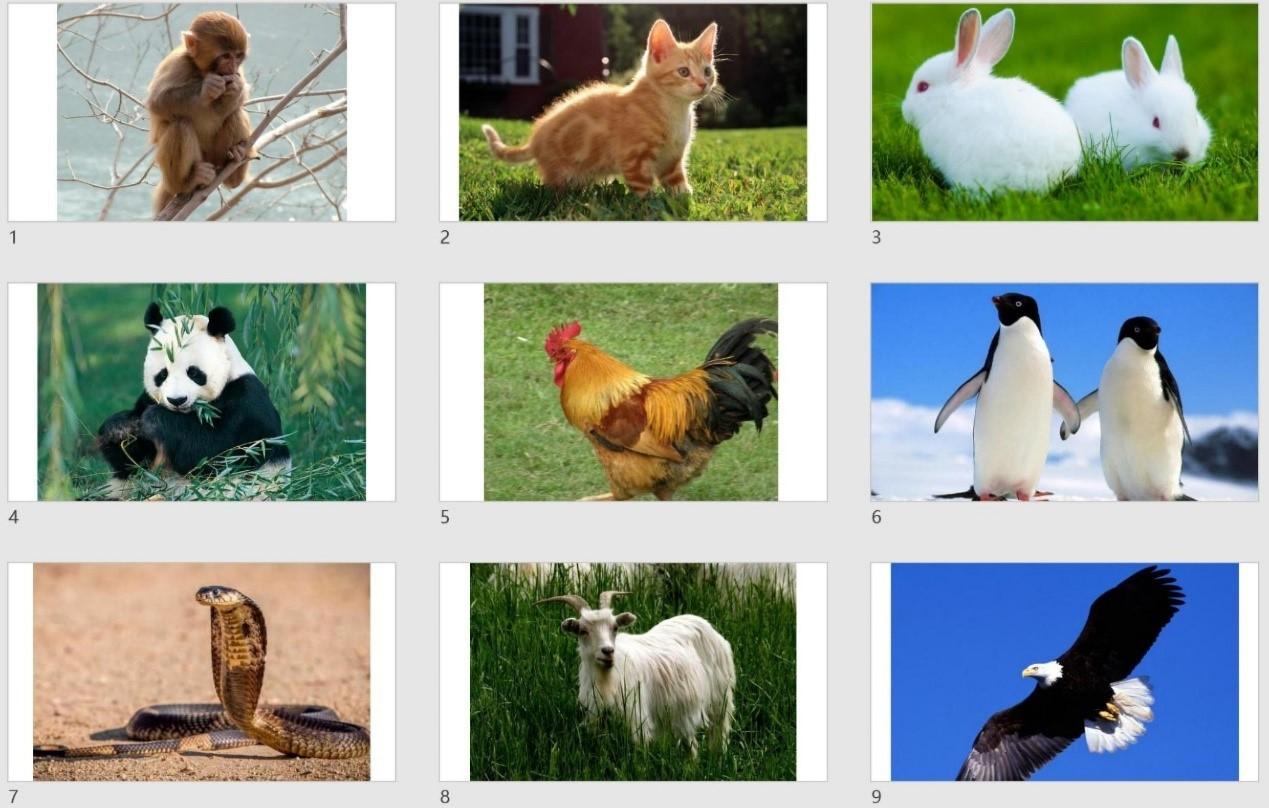


**Front**

**
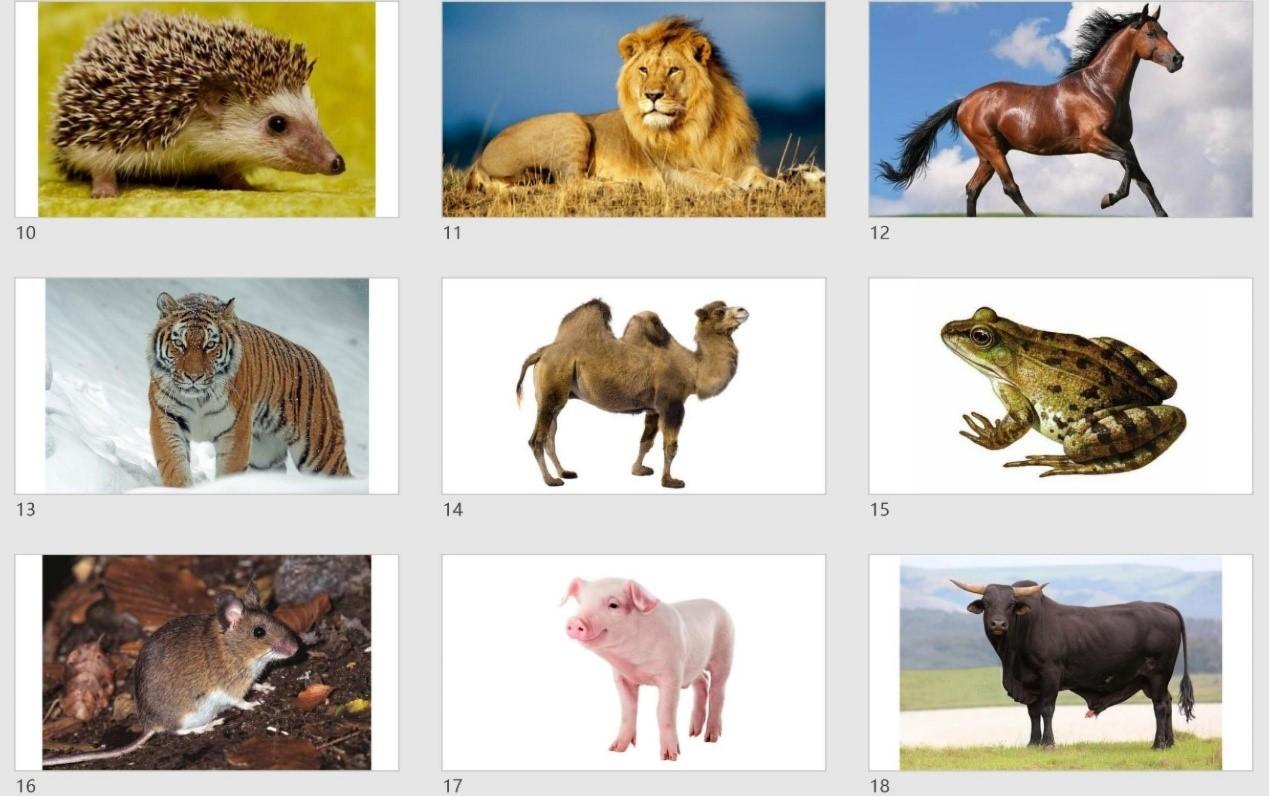
**

**Back**

**Supplementary Figure 1.** The A4 paper that prints the animals.
